# Small molecule induced reactivation of mutant p53 in cancer cells 

**DOI:** 10.1093/nar/gkt305

**Published:** 2013-04-27

**Authors:** Xiangrui Liu, Rainer Wilcken, Andreas C. Joerger, Irina S. Chuckowree, Jahangir Amin, John Spencer, Alan R. Fersht

**Affiliations:** ^1^MRC Laboratory of Molecular Biology, Francis Crick Avenue, Cambridge CB2 0QH, UK and ^2^Department of Chemistry, School of Life Sciences, University of Sussex, Falmer, Brighton, East Sussex BN1 9QJ, UK

## Abstract

The p53 cancer mutant Y220C is an excellent paradigm for rescuing the function of conformationally unstable p53 mutants because it has a unique surface crevice that can be targeted by small-molecule stabilizers. Here, we have identified a compound, PK7088, which is active *in vitro*: PK7088 bound to the mutant with a dissociation constant of 140 μM and raised its melting temperature, and we have determined the binding mode of a close structural analogue by X-ray crystallography. We showed that PK7088 is biologically active in cancer cells carrying the Y220C mutant by a battery of tests. PK7088 increased the amount of folded mutant protein with wild-type conformation, as monitored by immunofluorescence, and restored its transcriptional functions. It induced p53-Y220C-dependent growth inhibition, cell-cycle arrest and apoptosis. Most notably, PK7088 increased the expression levels of p21 and the proapoptotic NOXA protein. PK7088 worked synergistically with Nutlin-3 on up-regulating p21 expression, whereas Nutlin-3 on its own had no effect, consistent with its mechanism of action. PK7088 also restored non-transcriptional apoptotic functions of p53 by triggering nuclear export of BAX to the mitochondria. We suggest a set of criteria for assigning activation of p53.

## INTRODUCTION

The tumour suppressor p53 is a centrepiece in the cell’s defence against cancer (1). p53 is activated in response to cellular stresses and initiates a variety of cellular responses including cell cycle arrest and apoptosis (2,3). It is inactivated by mutation in many cancers, and loss of p53 function can lead to more aggressive cancer forms (4) or resistance to chemotherapy (5). Most p53 mutations are missense mutations in its DNA-binding (also called core) domain, which can be divided into two classes: contact mutations that interfere with p53-DNA contacts, and structural mutations that destabilize the protein, causing it to unfold and aggregate rapidly at physiological conditions (6). Analysis of the p53 mutation database of the International Agency for Research on Cancer (7) suggests that about 30–40% of p53 cancer mutations are structural mutations (8). In principle, temperature-sensitive destabilized structural mutants that are functional at low temperature but denature at higher temperatures may be rescued by small molecules that bind to the native state of p53 and stabilize it (9,10). Y220C is the ninth most frequent p53 cancer mutation and destabilizes the p53 core domain by about 4 kcal/mol, with the protein >80% unfolded at body temperature (10,11). Y220C is an ideal test case for a small-molecule stabilization approach because the tyrosine-to-cysteine mutation creates a unique surface crevice that is druggable. We have recently developed two classes of small molecules, substituted 9-ethylcarbazoles (12) and 2-(aminomethyl)-4-ethynyl-6-iodophenols (13), the latter featuring an iodine-oxygen halogen bond as core interaction.

Attempts to identify p53-activating compounds have been made from antibody assays and cell-based screening (14). The first of these compounds, CP-31398, (15) was reported to stabilize the p53 core domain and be active against cancer xenografts. But, it was later shown using direct biophysical techniques that CP-31398 does not bind to the p53 core domain, does not stabilize p53 *in vitro* but is a DNA intercalator (16) and is toxic to cell lines H1299 and Saos-2, which are commonly used to test p53 activity (17). It is our experience in conducting many cell-based assays that there is a large number of false positives for the reactivation of p53 mutants that somehow result from more general effects of cytotoxicity or more specific effects that appear to manifest themselves as p53 reactivation.

Here, we have combined biophysical and cell-based techniques to identify and characterize a new compound, PK7088. This compound has been identified from an in-house synthesized fragment library (18–23), binds to the p53 Y220C core domain and, most importantly, reactivates cellular p53 functions in p53-Y220C mutant cells.

## MATERIALS AND METHODS

### Protein NMR spectroscopy

^1^H/^15^N-heteronuclear single quantum coherence (HSQC) spectra of uniformly ^15^N-labelled T-p53-Y220C (75 µM) with and without PK7088 were acquired at 20°C on a Bruker Avance-800 spectrometer using a 5-mm inverse cryogenic probe. Samples were prepared by adding dilutions of compound from stock solutions in DMSO-d_6_ to a final concentration of 5% (v/v) DMSO-d_6_ in buffer. All HSQC spectra were acquired with 8 transients per t_1_ data point, 1024 data points in t_2_, and 64 complex data points in t_1_, with spectral widths of 11.0 kHz for ^1^H and 2.7 kHz for ^15^N, and a recycle delay of 800 ms. Chemical shifts were considered significant if the average weighted ^1^H/^15^N chemical shift difference 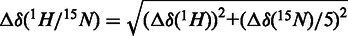 was greater than 0.04 ppm. To determine dissociation constants, at least five ^15^N/^1^H HSQC spectra at different compound concentrations were measured. Spectra analysis was performed using Sparky 3.114 (24) and Bruker Topspin 2.0 software. To derive K_D_ values, a quadratic saturation binding equation was fitted to the concentration-dependent chemical shift changes of the relevant shifting peaks:

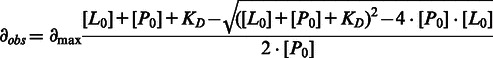



### Differential Scanning Fluorimetry (DSF)

The effect of PK7088 on the melting temperature of T-p53C-Y220C was measured using SYPRO Orange as described previously (11). PK7088 was added to the protein at a range of concentrations (75, 150, 250 and 350 μM).

### X-ray crystallography

Crystals of T-p53C-Y220C were grown as described previously (11). They were soaked for 3 h in a 40 mM solution of compound PK7242 in 19% polyethylene glycol 4000, 20% glycerol, 10 mM sodium phosphate, pH 7.2, 100 mM HEPES, pH 7.2, 150 mM KCl and 10 mM dithiothreitol (DTT), and flash frozen in liquid nitrogen. An X-ray data set was collected at 100 K on beamline I02 at the Diamond Light Source, Oxford. The data set was processed with XDS (25) and SCALA (26). The structure was solved by rigid body refinement with PHENIX (27) using the structure of the ligand-free mutant (PDB ID 2J1X) as a starting model. Iterative model building and refinement was done using Coot (28) and PHENIX. Data collection and refinement statistics are shown in Supplementary Table S2. The atomic coordinates and structure factors of the Y220C-PK7242 complex have been deposited in the Protein Data Bank, www.pdb.org (PDB ID code 3ZME). Structural figures were prepared using PyMOL (www.pymol.org).

### Cell culture

HUH-7 (p53-Y220C^+/+^, registration no. JCRB0822), HUH-6 (wild-type p53^+/+^, registration no. JCRB0834), NUGC-3 (p53-Y220C^+/+^, registration no. JCRB0401), NUGC-4 (wild-type p53^+/+^, registration no. JCRB0403) and MKN-1 (p53-V143A^+/+^, registration no. JCRB0252) cells were purchased from Japan Health Science Research Resources Bank. HUH-7 and HUH-6 cells were maintained in DMEM medium with 10% fetal calf serum and 1% antibiotic stock mix (10 000 U/ml penicillin, 10 000 μg/ml streptomycin), and other cell lines were maintained in RPMI1640 medium with the same concentration of serum and antibiotics. All cells were incubated in a humidified incubator at 37°C with 5% CO_2_.

### Cell viability assay

Cell viability was monitored by crystal violet staining. Briefly, 1 × 10^5^ cells were seeded per well in 6-well plates. After 24-h treatment, cells were washed with PBS and fixed using 50:50 methanol/acetone solution (v/v). Cells were then stained with crystal violet (0.2% w/v in 2% ethanol) for 30 min, washed with PBS and dried at room temperature.

### Caspase-3/7 assay

Activity of caspase 3/7 was measured using a Caspase-Glo 3/7 assay kit (Promega G8091), following the manufacturer’s instructions. PRIMA-1^MET^ was purchased from Santa Cruz Biotechnology.

### p53 knockdown by siRNA

Silencing of p53 was achieved by transfection of human-specific p53 siRNA (Cell Signaling Technology) using the RiboJuice siRNA Transfection Reagent (Novagen). Downregulation of p53 expression was confirmed by western blots.

### Cell cycle analysis

Cells were treated for 6 h, collected and re-suspended in 0.4 ml hypotonic fluorochrome solution and analysed with an Eclipse Flow Cytometry Analyzer. Nutlin3 was purchased from Tocris Bioscience.

### Annexin V/PI staining

AnnexinV/propidium iodide (PI) staining was used to determine the percentage of apoptotic cells after treatment for 24 h. The pan-caspase inhibitor Z-Val-Ala-Asp-(OMe)-CH2F (Z-VAD-fmk, Sigma) was used at a concentration of 50 μM. Sample preparation, staining and analysis were performed following the protocol provided by BD Bioscience.

### Western blots

Western blotting was performed as described (29). The following antibodies were used: p53 Do-7 (Dako), p21 (Millipore), Puma (Abcam), Noxa (Abcam), MDM2 (Abcam), Bax (Cell Signalling Technologies), β-actin (Abcam). Both anti-mouse and anti-rabbit immunoglobulin G (IgG) horseradish peroxidase-conjugated antibodies were obtained from Dako.

### Real-Time PCR

HUH-7 cells were treated with 200 μM PK7088 or DMSO control for 6 h. RNA was extracted and purified using RNeasy Mini Kit (QIAGEN) and RNase-Free DNase Set (QIAGEN). cDNA reverse transcription was performed using High Capacity RNA-to-cDNA Kit (Applied Biosystems). Twenty nanogram cDNA and 10 μl of TaqMan Gene Expression Master Mix (Applied Biosystems) were added to each well of a TaqMan Human Cellular apoptosis Pathway plate (Applied Biosystems). The thermal-cycling conditions were set according to the manufacturer’s instructions, and reactions were performed using a ViiA™ 7 Real-Time PCR System (Applied Biosystems). The comparative threshold cycle (Ct) method was used to analyse the gene expression levels.

### Immunofluorescence

Cells were treated with 200 μM PK7088 or DMSO control for 4 h or 6 h, fixed and permeabilized using 50:50 methanol/acetone solution (v:v). The following antibodies were used: anti-p53 antibody Pab 1620 (Abcam), anti-p53 antibody Pab 240 (Abcam), anti-Bax (Cell signalling) and goat Pab to Ms Dylight 488 (Abcam). Hoechst 33342 (cell signalling) and MitoTracker® Red CMxRos (Lonza) were used to stain the nucleus and mitochondria of cells. Imaging was performed using a Zeiss710 system.

## RESULTS

### PK7088 binds and stabilizes the Y220C mutant

**Figure 1.**
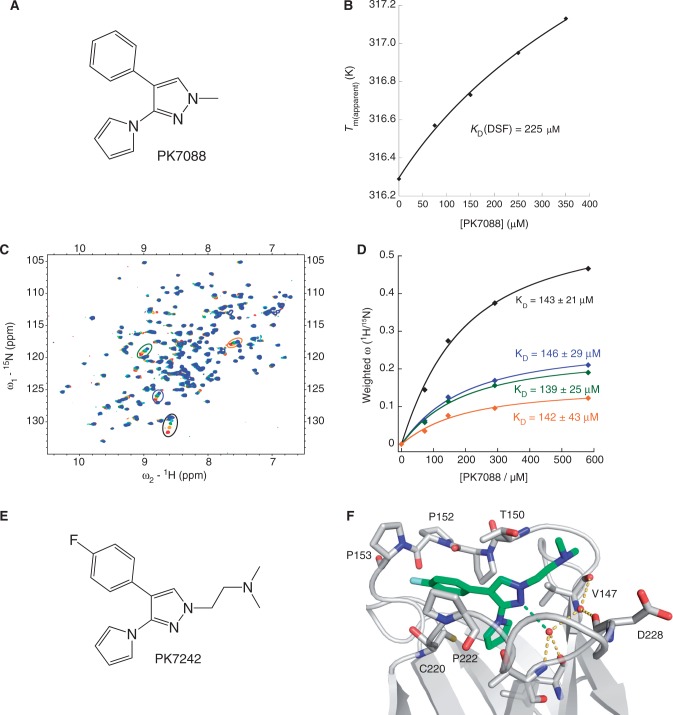
Biophysical and structural characterization of PK7088 binding to p53-Y220C. (**A**) Chemical formula of PK7088. (**B**) Concentration-dependent thermostabilization of the Y220C mutant by PK7088 measured by DSF. (**C**) Overlay of ^1^H/^15^N-HSQC NMR spectra of p53-Y220C core domain (94-312) with varying concentrations of PK7088. (**D**) Quadratic saturation binding equation fitted to the concentration-dependent chemical shift changes of the relevant shifting peaks yields a *K*_D_ ≈ 140 µM. (**E**) Chemical formula of PK7242, a more soluble analogue of PK7088 that was used for crystallography. (**F**) Crystal structure of p53-Y220C core domain in complex with PK7242. The binding pocket is depicted as a grey ribbon diagram, with selected side chains shown as stick models. PK7242 is shown as a green stick model. The interaction of the central pyrazole moiety with a network of structural water molecules is highlighted with broken lines.

The small molecule 1-methyl-4-phenyl-3 -(*1H*-pyrrol-1-yl)-*1H*-pyrazole (PK7088, Figure 1A) was identified as a Y220C binder by protein-observed NMR screening and binds to this mutant with *K*_D_ = 140 μM as determined by chemical shift mapping using ^1^H/^15^N-HSQC NMR spectroscopy (Figure 1C and D). It also stabilizes p53-Y220C core domain by ∼1 K at 350 μM compound concentration (Figure 1B). Attempts to solve the crystal structure of the Y220C–PK7088 complex failed because of the relatively poor solubility of the molecule. We designed, therefore, a more soluble derivative of PK7088, PK7242, by adding an *N*,*N*-dimethyl-ethanamine linker (Figure 1E). ^1^H/^15^N-HSQC spectra of p53-Y220C core domain in the presence of PK7088 or PK7242 were virtually identical, indicating that both compounds have the same overall binding mode (Supplementary Figure S1). Compound PK7242 was soaked into Y220C crystals, and the crystal structure of the complex was solved at 1.35 Å resolution, with excellent electron density for the ligand. The binding mode is shown in Figure 1F. Binding of PK7242 is accompanied by a flip of the Cys220 side chain, which increases the depth of the cavity to accommodate the pyrrole moiety. The central pyrazole ring is sandwiched between Val147, Thr150 and Pro151 on one side and Pro222 and Pro223 on the other side of the pocket. It forms a hydrogen bond with a structural water molecule stabilized by the backbones of Leu145 and Thr230. The fluorophenyl moiety points toward a subsite that was previously targeted via an acetylene linker (13). The *N*,*N*-dimethyl-ethanamine linker, added to increase the solubility of the compound, is solvent-exposed.

### PK7088 induces caspase-3/7 selectively in p53-Y220C cells

**Figure 2.**
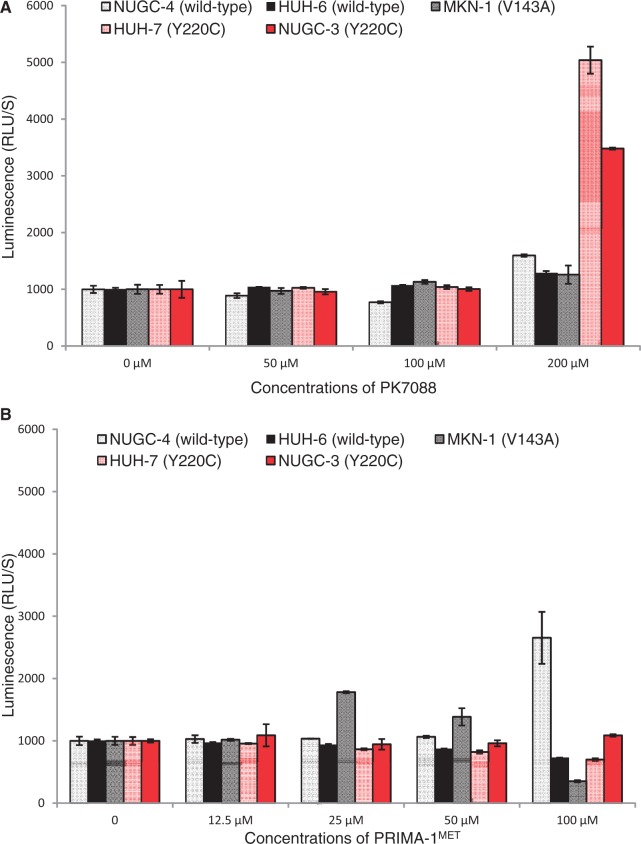
Induction of caspase 3/7 by PK7088 or PRIMA-1. PK7088 activates caspase 3/7 selectively in p53-Y220C mutant cells (**A**), while PRIMA-1 induced caspase in p53-V143A mutant cells and p53 wild-type cells (**B**). Induction of caspase 3/7 activity was determined after 6-h PK7088 treatment in five cell lines with different p53 status, including the Y220C mutant (NUGC-3, HUH-7), the V143A mutant (MKN-1) and wild-type p53 (NUGC-4, HUH-6).

Apoptotic effects of PK7088 on three gastric cancer cell lines (NUGC-3, NUGC-4 and MKN-1) and two human hepatoblastoma cell lines (HUH-6 and HUH-7) were determined by induction of caspase-3/7 after 6 h PK7088 treatment at different concentrations (Figure 2). These cell lines were chosen because they have a different p53 status with homozygous p53 gene (Y220C, V143A or wild-type). Compared with DMSO control, PK7088 induced caspase-3/7 significantly at 200 μM in the Y220C-containing NUGC-3 and HUH-7 cells, while only small effects were observed in p53 wild-type (NUGC-4, HUH-6) and V143A mutant (MKN-1) cells. We also studied the effects of Prima-1^MET^, a reported mutant p53 reactivator, on caspase induction (Figure 2B). At 25 μM compound concentration, Prima-1^MET^ increased caspase-3/7 activity only in MKN-1 cells. On treatment with 100 μM Prima-1^MET^, induction of caspase-3/7 was also observed in NUGC-4 cells, whereas Prima-1^MET^ had no effect in p53-Y220C cell lines at the concentrations tested.

### PK7088 restores wild-type p53 conformation in p53-Y220C cells

**Figure 3.**
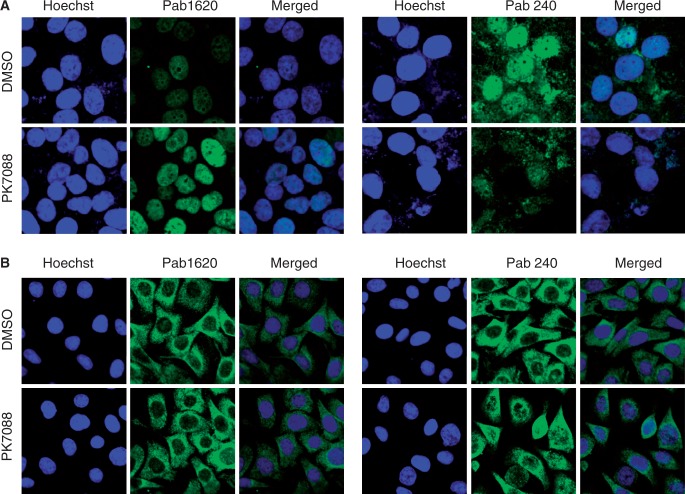
Effect of PK7088 on the folding state of p53 in different cancer cell lines. PK7088 increased the amount of correctly folded p53 while decreasing the amount of unfolded p53 in HUH-7 (p53-Y220C) cells but not MKN-1 (p53-V143A) cells. Immunostaining of HUH-7 cells (**A**) and MKN-1 cells (**B**) was performed using the conformation-specific antibodies Pab1620 (wild-type/folded) and Pab240 (mutant/unfolded). Both cell lines were treated with DMSO control or 200 μM PK7088 for 4 h. Hoechst 33342 dye was used to stain the nucleus.

To investigate whether PK7088 stabilizes the Y220C mutant in cancer cells and increases the amount of correctly folded p53 protein, we performed immunofluorescence assays using two conformation-specific antibodies, PAb240 and PAb1620, after 4 h incubation with the small molecule. The PAb240 antibody recognizes an epitope that is buried in the hydrophobic core of the protein (i.e. it can only bind to unfolded p53) (30,31), whereas PAb1620 binds an epitope on the surface of correctly folded p53 (32). In HUH-7 cells, PK7088 significantly increased the amount of folded Y220C mutant, as detected with Pab1620, showing a 76% increase in fluorescence intensity compared with untreated cells after normalization. In contrast, the fluorescence intensity of antibody PAb240 (detecting unfolded protein) was decreased to 38% of that in untreated cells, suggesting an almost 3-fold reduction in the amount of unfolded mutant (Figure 3A and Supplementary Figure S2). In addition, the folded protein was found mainly in the nucleus, while mutant p53 was observed both inside and outside the nucleus of untreated HUH-7 cells. In contrast, PK7088 had no effect on the folding state of p53 in V143A mutant MKN-1 cells (Figure 3B).

### PK7088 induces p53-Y220C-dependent cell cycle and apoptotic effects

**Figure 4.**
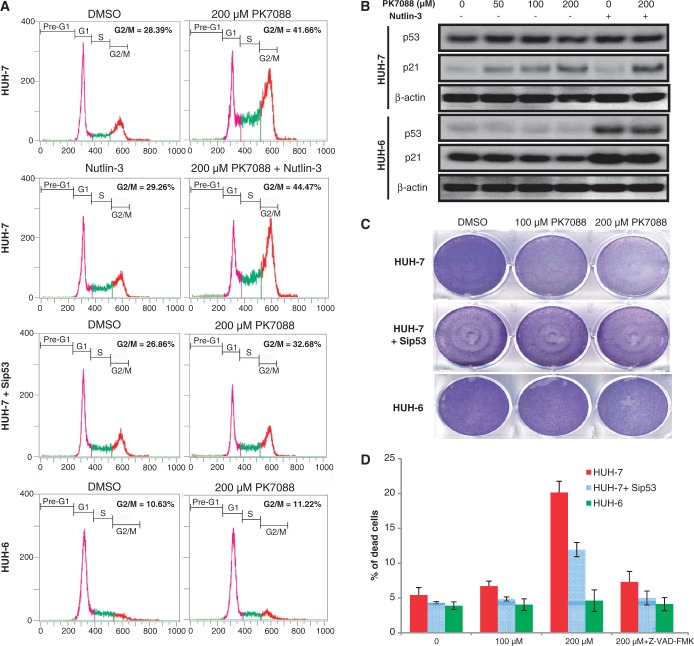
Y220C mutant-specific cell-cycle and apoptotic effects of PK7088. (**A**) FACS analysis of HUH-7 and HUH-6 cells after 6-h incubation with 200 μM PK7088. PK7088 induced p53-dependent G2/M phase arrest in HUH-7 cells, which was enhanced by Nutlin-3. (**B**) Protein levels of the p53 target gene p21 detected by western blots after 6-h treatment. PK7088 induced p21 in HUH-7 cells but not HUH-6 cells. Nutlin-3 induced p21 in p53 wild-type HUH-6 cells only, but worked synergistically with PK7088 in up-regulating p21 in HUH-7 cells. (**C**) Crystal violet staining was used to determine the cell viability of HUH-7 and HUH-6 cells after 24-h treatment. PK7088 decreased the cell viability. This effect was counteracted by p53 knockdown. Only very limited effects were observed in HUH-6 cells. (**D**) HUH-7 and HUH-6 cells were exposed to PK7088 for 24 h and analysed by AnnexinV/PI staining. In HUH-7 cells, 200 μM PK7088 induced apoptosis, which was counteracted by p53 knockdown. No cell death was detected in HUH-6 cells. The pan-caspase inhibitor Z-VAD-fmk (50 μM) blocked the cell death effect induced by PK7088.

p53 silencing was performed in HUH-7 cells to confirm the p53-Y220C-specific effects of PK7088. The MDM2 antagonist Nutlin-3, which activates the p53 pathway in p53 wild-type cells, was used to confirm restoration of p53 function after PK7088 treatment. In HUH-7 cells, p53 knockdown or Nutlin-3 (10 μM) had no notable effect on the cell cycle (Figure 4A). Incubation with 200 μM PK7088 for 6 h caused G_2_/M cell-cycle arrest, which was increased further when combined with treatment with 10 μM Nutlin-3. In p53-silenced HUH-7 cells, the percentage of cells arrested in the G_2_/M phase was reduced considerably from 42% to 32%. In contrast, PK7088 did not induce G_2_/M arrest in HUH-6 cells.

To study further the p53-Y220C-dependent cell-cycle effect of PK7088, we determined the protein level of the p53 target gene p21 by western blotting (Figure 4B). PK7088 induced p21 expression after 6-h treatment in HUH-7 but not HUH-6 cells. As expected, Nutlin-3 increased p21 only in p53 wild-type HUH-6 cells but did not change the p21 level in mutant HUH-7 cells. In the presence of PK7088, Nutlin-3 enhanced the up-regulation of p21 only in HUH-7 cells but not in HUH-6 cells.

The cytotoxic and apoptotic effects of PK7088 were determined by crystal violet (Figure 4C) and AnnexinV/PI staining (Figure 4D), respectively. PK7088 reduced the cell viability of HUH-7 cells at 100 μM after 24-h treatment, whereas p53-silenced cells were less sensitive to PK7088. In HUH-7 cells, induction of apoptosis was detected after 24-h incubation with 200 μM PK7088. p53 knockdown reduced the cell death caused by PK7088. In the presence of the pan-caspase inhibitor Z-VAD-fmk (50 μM), the apoptotic effect of PK7088 was largely inhibited. HUH-6 cells were insensitive to PK7088 at the tested ligand concentrations.

### PK7088 induces NOXA and regulates Bax relocation

**Figure 5.**
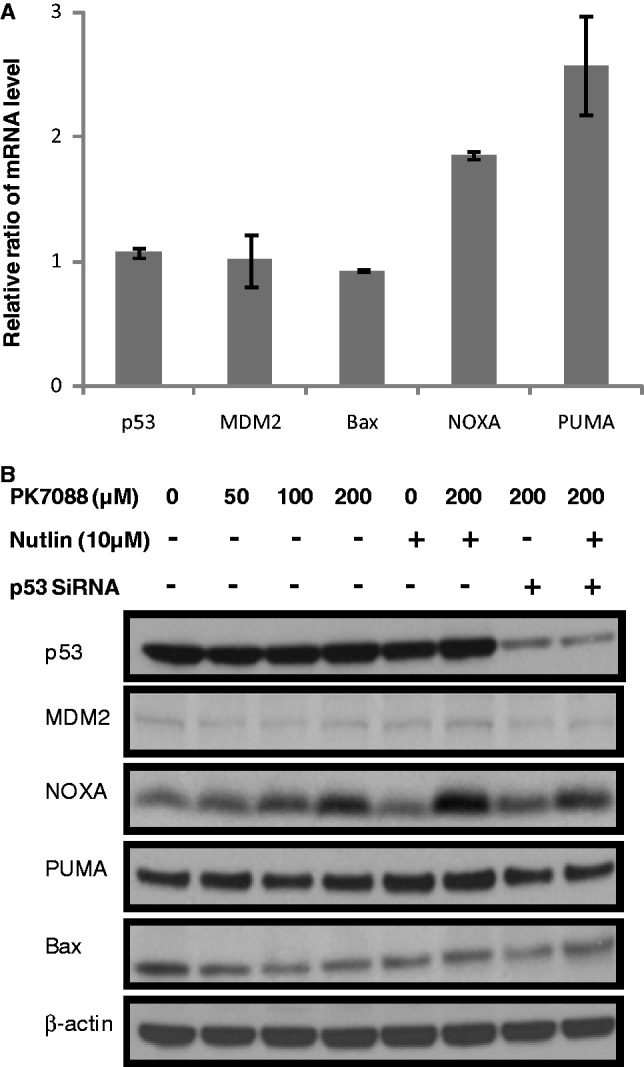
Restoration of wild-type p53 functions in HUH-7 cells by PK7088. (**A**) mRNA levels of p53 target genes measured by real-time PCR after 6-h treatment. 200 μm PK7088 up-regulated NOXA and PUMA mRNA, but had no effect on p53, MDM2 or Bax. (**B**) Protein levels of p53 target genes determined by western blots. PK7088 induced dose-dependent up-regulation of NOXA protein, whereas PUMA or Bax levels were unaffected.

**Figure 6.**
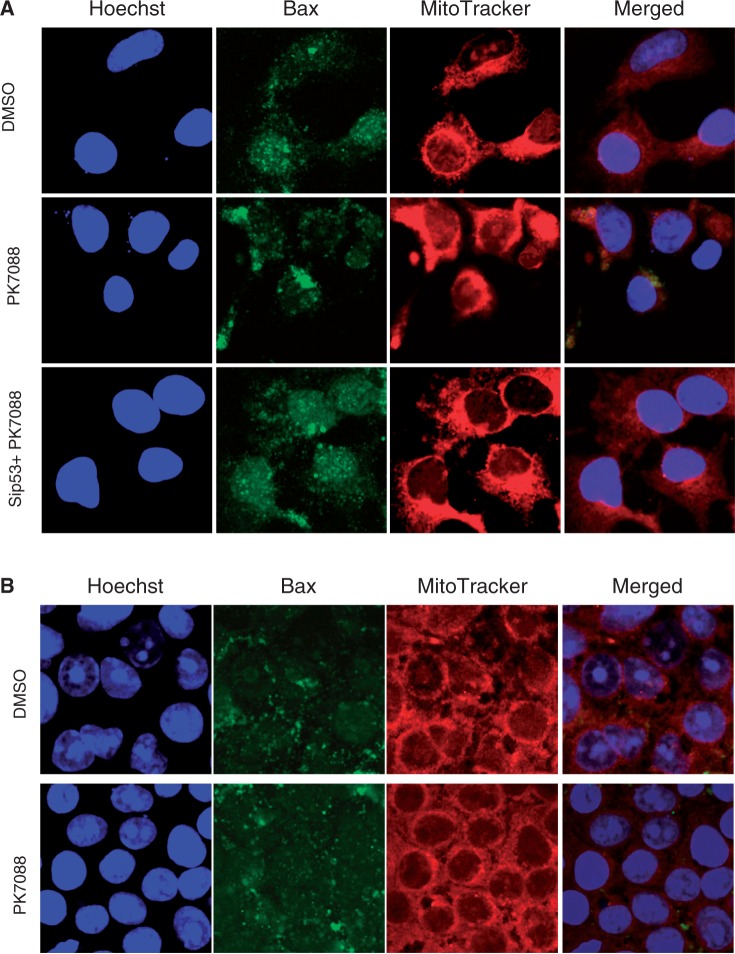
Effect of PK7088 on the cellular localization of Bax. PK7088 induced relocation of Bax to the mitochondria in HUH-7 cells (**A**), but not in HUH-6 cells (**B**) after 6-h treatment at 200 μm. Nucleus and mitochondria were stained with Hoechst 33342 and MitoTracker Red CMxRos dye, respectively.

The mRNA levels of p53, MDM2 and the pro-apoptotic proteins Bax, NOXA and PUMA in HUH-7 cells were measured by real time PCR after 6-h incubation with 200 µM PK7088 (Figure 5A). NOXA and PUMA mRNA levels were increased 1.9- and 2.6-fold, respectively, while the mRNA levels of MDM2 and Bax remained unchanged. As shown in Figure 5B, western blots confirmed that the majority of p53 was knocked down by siRNA, and PK7088 increased the expression of NOXA in a p53-dependent manner. Nutlin-3 alone had no effect on NOXA expression, but it further increased NOXA expression in the presence of PK7088. Little effect was observed on MDM2, PUMA or Bax protein levels. Interestingly, we observed p53-dependent relocation of Bax to the mitochondria after treating HUH-7 cells for 6 h with 200 μM PK7088 (Figure 6A). Hoechst dye (blue) was used to visualize the location of the nucleus, while the mitochondria of HUH-7 cells were stained with MitoTracker dye (red). In untreated HUH-7 cells, Bax was located mainly in the nucleus, whereas PK7088 seemed to induce the translocation of Bax from the nucleus to the mitochondria. In HUH-7 cells where p53 had been knocked down, the relocation of Bax was inconspicuous compared with un-silenced cells. In addition, the distribution of Bax was unaffected in wild-type HUH-6 cells (Figure 6B).

### PK7088 does not inhibit a range of cancer kinases

To rule out off-target kinase inhibitory effects contributing to the cellular activity of PK7088, we screened this compound as well as its demethylated analogue PK7209, which does not stabilize Y220C, against a panel of 74 cancer kinases at a concentration of 50 µM. No significant effects were observed (Supplementary Table S1). Also, PK7088 does not inhibit b-raf kinase although its chemical structure resembles that of some reported b-raf inhibitors (33).

## DISCUSSION

With its unique surface crevice, the p53 cancer mutant Y220C is an excellent test case for the development of p53 rescue drugs. We have previously developed different classes of small-molecule stabilizers of this mutant and extensively analysed their binding mode and their effect on melting temperature and aggregation properties (12,13,34,35). Here, we have discovered a new class of compounds based on a substituted 4-phenyl-3-(*1H*-pyrrol-1-yl)-*1H*-pyrazole scaffold and determined the binding mode of a representative member of this compound class by X-ray crystallography, revealing key interactions with the Y220C-specific binding pocket.

The most interesting aspect of the small molecule PK7088, which has a moderate binding constant of 140 µM *in vitro*, is its potency in cancer cell lines carrying the Y220C mutation. Our immunofluorescence assays using the p53-conformation-specific antibodies PAb1620 and PAb240 to monitor the folding state of p53 clearly showed that PK7088 significantly increases the amount of correctly folded p53 in HUH-7 cells, thus potentially restoring p53 function. Consistent with the proposed mechanism of action of PK7088 as mutant-specific small-molecule stabilizer, no effect on the levels of folded p53 protein was observed in cancer cell lines with V143A mutation. A similar increase in the amount of folded mutant protein in a cancer cell line induced by a small molecule was recently reported for the structural mutant R175H which, like Y220C, is normally largely unfolded at body temperature (36). Given the structure of the Y220C mutant (11) and its reported temperature-sensitive phenotype (37,38), an increase in the amount of folded protein in the HUH-7 cancer cells should result in the up-regulation of p53 target genes. Indeed, we observed up-regulation of various p53 downstream targets both at the mRNA and protein level upon PK7088 treatment in a mutant-specific manner, affecting both cell-cycle arrest and apoptotic functions.

To assess the cell-cycle arrest function of p53, we monitored the protein levels of the transcriptional target p21, which inhibits cyclin-dependent kinases (CDKs) and causes cell-cycle arrest. Up-regulation of p21 by a tetracycline-regulated system, for example, induces G_2_ arrest in many cell lines, including Hela, Saos-2, U2OS and H1299 (39). We found that PK7088 induces p21- and p53-dependent G_2_/M arrest in p53-Y220C cells but not p53 wild-type cells, suggesting restoration of wild-type p53 function of the Y220C mutant in the HUH-7 cells. Interestingly, PK7088 acted synergistically with Nutlin-3. Nutlin-3 mimics the transactivation domain of p53 and competes with binding of the latter to the N-terminal domain of MDM2, thereby inhibiting MDM2-mediated degradation of p53 (40). Nutlin-3-induced effects, such as cell-cycle arrest and induction of apoptosis, are therefore generally only observed in cancer cell lines with wild-type p53, but not in cells that carry inactive mutant p53 (41–43). As expected, Nutlin-3 had no effect on HUH-7 cells in the absence of PK7088. When combined with PK7088 treatment, however, Nutlin-3 increased p21 expression and G_2_/M arrest of HUH-7 cells, indicating levels of active p53 that are further up-regulated through inhibition of one of its degradation pathways.

In addition to the above effects on cell-cycle arrest, we observed several apoptosis-related events upon PK7088 treatment that were specific to HUH-7 (p53-Y220C) cancer cells. As part of the p53-mediated apoptotic process, caspases undergo proteolytic activation (44). Apoptosis and caspase 3/7 activity were induced selectively in p53-Y220C HUH-7 cells upon treatment with 200 uM PK7088. This induction of apoptosis was inhibited by addition of pan caspase inhibitor Z-VAD-fmk, indicating that PK7088 triggers apoptosis in a p53-Y220C- and caspase-dependent manner. We also observed up-regulation of mRNA levels of the p53 downstream targets PUMA and NOXA, two critical mediators of the p53-induced apoptotic responses (45,46), after 6-h incubation with PK7088. NOXA protein levels were also increased whereas no effect on the protein levels of PUMA was observed. Importantly, both mRNA and total p53 protein level were unchanged, indicating that PK7088 works by increasing the ratio of correctly folded Y220C mutant at a given expression level. PK7088 binds non-covalently to Y220C and acts in an entirely different way from Prima-1^MET^, which binds covalently to the p53 core domain via alkylation of cysteine residues (47), which affects DNA binding (48). Accordingly, the precise reactivation mechanism of mutant p53 by Prima-1^MET^ is different from that of 7088.

p53 can promote apoptosis through transcription-independent mechanisms (49,50). The crucial step of the transcription-independent pathway in p53-mediated apoptosis is the stress-induced accumulation of p53 in the cytosol or mitochondria, leading to activation of Bax or Bak (3,49). During apoptosis, Bax is translocated from the cytosol to mitochondria where it triggers mitochondrial outer membrane permeabilization (51), and wild-type p53 was reported to activate Bax directly (52) and induce a conformational change in Bax (53). Intriguingly, we observed translocation of folded p53 from the nucleus to the cytoplasm after 4-h treatment with PK7088 in HUH-7 cells. The mRNA and protein levels of Bax were unaffected by PK7088, but PK7088-induced relocation of Bax to the mitochondria was observed, which was prevented by p53 knockdown, providing further evidence that PK7088 restores various aspects of p53 function.

### Criteria for reactivation of p53 in cell lines

As we have found several molecules that induced the p53 target gene p21 in cell-based screening assays but showed no detectable binding to Y220C *in vitro*, we suggest the use of the following criteria to screen for genuine p53 reactivation: direct demonstration of binding to the mutant protein and its thermal stabilization; visualization in cell lines that p53 is restored to its folded state in cells by using antibodies such as PAb1620 and PAb240; and activation of p53 target genes such as p21 that is abolished on p53 knockdown, or equivalent mutant-specific induction in isogenic cell lines differing only in p53 mutation status. p53-dependent synergistic effects of inhibitors of MDM2 on transcription provide further evidence.

In fulfilling all these criteria, we have proven in this study that PK7088 is a mutant-specific p53-Y220C stabilizer. Future structure-guided optimization of the current lead should increase its affinity to the Y220C mutant and further improve its selectivity in cancer cells.

## SUPPLEMENTARY DATA

Supplementary Data are available at NAR Online: Supplementary Tables 1–2 and Supplementary Figures 1–2.

## FUNDING

ERC Advanced Grant ‘Tumour suppressor p53: structure, stability and novel anti-cancer drug development’ [P53LAZARUS]; EPSRC-BBSRC-MRC Collaborative Network in Chemical Biology (SMSdrug.net) (to J.S.). Funding for open access charge: European Research Council Grant.

*Conflict of interest statement*. None declared.

## Supplementary Material

Supplementary Data
