# Supplementary material for: Small molecule induced reactivation of mutant p53 in cancer cells
Source: Nucleic Acids Res. 2013 Apr 27;41(12):6034–44. doi: 10.1093/nar/gkt305 (PMC3695503; doi:10.1093/nar/gkt305)
Supplement: Supplementary Data [file supp_41_12_6034__index.html]

Small molecule induced reactivation of mutant p53 in cancer cells — Small molecule induced reactivation of mutant p53 in cancer cells — Supplementary Data 

# Small molecule induced reactivation of mutant p53 in cancer cells

## Supplementary Data

files

**Files in this Data Supplement:**

- Supplementary Data - doc file
