# Supplementary material for: Small molecule induced reactivation of mutant p53 in cancer cells
Source: Nucleic Acids Res. 2013 Apr 27;41(12):6034–44. doi: 10.1093/nar/gkt305 (PMC3695503; doi:10.1093/nar/gkt305)
Supplement: Supplementary Data [file supp_gkt305_nar-00517-h-2013-File008.doc]

**Small-molecule induced reactivation of mutant p53 in cancer cells**

Xiangrui Liu^1^, Rainer Wilcken^1^, Andreas C. Joerger^1^, Irina S. Chuckowree^2^, Jahangir Amin^2^, John Spencer^2^ and Alan R. Fersht^1*^

^1^ MRC Laboratory of Molecular Biology, Hills Road, Cambridge CB2 0QH, United Kingdom.

^2^ Department of Chemistry, School of Life Sciences, University of Sussex, Falmer, Brighton, East Sussex, BN1 9QJ, United Kingdom

* To whom correspondence should be addressed. Tel: +44‐1223‐40‐2137, Fax: +44‐1223‐40‐2140. E‐mail: [arf25@cam.ac.uk](mailto:arf25@cam.ac.uk).

Present Address: [Author name], Department, Institution, Town, State, Postcode, Country

**SUPPLEMENTARY DATA**

**Chemical compounds**

**1-Methyl-4-phenyl-3-(*1H*-pyrrol-1-yl)-*1H*-pyrazole (PK7088)**

1-Methyl-4-phenyl-5-aminopyrazole (500 mg, 2.89 mmol) and 2,5-dimethoxytetrahydrofuran (1.1 equiv., 0.42 mL) were heated in acetic acid (5 ml) and the reaction mixture was stirred under microwave irradiation at 120 ^o^C for 15 min (maximum power 200 W) in a CEM Explorer. The mixture was cooled to room temperature and volatiles were removed *in vacuo.* CH_2_Cl_2_ extraction was followed by an aq.Na_2_CO_3_ wash. After drying (MgSO_4_), purification using an ISCO CombiFlash purification unit (gradient 0-10% MeOH in CH_2_Cl_2_) gave the expected product as clear oil (415 mg, 65%). ^1^H NMR (CDCl_3_) δ: 7.47 (1H, s), 7.32-7.22 (3H, m), 7.15-7.12 (2H, m), 6.85 (2H, t, *J* 2.7 Hz), 6.24 (2H, t, *J* 2.7 Hz), 3.94 (3H, s); ^13^C NMR (CDCl_3_) δ: 145.8, 131.2, 130.0, 128.7 (2C), 127.6 (2C), 127.0, 120.9 (2C), 114.9, 109. 5 (2C), 39.4; MS (ESI, MH^+^) *m/z* 224.1 (100%); HRMS (ESI, MH^+^) *m/z* 224.1209 (calcd for C_14_H_14_N_3_ 224.1188).

**2-(4-(4-Fluorophenyl)-3-(*1H*-pyrrol-1-yl)-*1H*-pyrazol-1-yl)-*N,N*-dimethylethanamine (PK7243) and 2-(4-(4-fluorophenyl)-5-(*1H*-pyrrol-1-yl)-*1H*-pyrazol-1-yl)-*N,N*-dimethylethanamine (PK7242)**

4-(4-Fluorophenyl)-3-(*1H*-pyrrol-1-yl)-*1H*-pyrazole (224 mg, 1.1 mmol, made as above and used without further purification) was treated with NaH (56 mg, 1.4 mmol of a 60% suspension in oil) and left to stir at room temperature for 2h in anhydrous DMF (10 ml). Next 2-chloro-N,N-dimethylethanamine.HCl (172 mg, 1.2 mmol) and one more portion of NaH (56 mg, 1.4 mmol) were added and the reaction mixture was stirred overnight at rt. to afford a ca. 5:1 mixture of products after the usual work-up (ethyl acetate extraction, 3 brine washes, drying (MgSO_4_). Purification using an ISCO CombiFlash purification unit (gradient 0-100% acetone in EtOAc) afforded the regioisomeric products 2-(4-(4-fluorophenyl)-5-(*1H*-pyrrol-1-yl)-*1H*-pyrazol-1-yl)-*N,N*-dimethylethanamine (40 mg, 12% yield. Rf=0.3) and 2-(4-(4-fluorophenyl)-3-(*1H*-pyrrol-1-yl)-*1H*-pyrazol-1-yl)-*N,N*-dimethylethanamine (108 mg, 33% yield. Rf=0.25, neat acetone) as oils.

Minor (PK7243): 2-(4-(4-fluorophenyl)-5-(*1H*-pyrrol-1-yl)-*1H*-pyrazol-1-yl)-*N,N*-dimethylethanamine (contaminated with ca. 10% of regiomer): ^1^H NMR (CDCl_3_) δ: 7.79 (1H, s), 7.00-6.92 (4H, m), 6.74 (2H, d, J= 2.0 Hz), 6.40 (2H, d, J= 2.0 Hz), 3.98 (2H, t, J= 6.8 Hz), 2.68 (2H, d, J = 6.8 Hz), 2.32 (6H, s). ^13^C NMR (CDCl_3_) δ: 161.6 (d, ^1^J_CF_ =239 Hz), 136.5, 135.3, 127.1, 127.0, 119.8, 116.6, 115.7, 112.3, 58.8, 46.5, 45.5. HRMS (ESI, MH^+^) *m/z* 299.1665 (calcd for C_17_H_20_FN_4_ 299.1667).

Major (PK7242): 2-(4-(4-fluorophenyl)-5-(*1H*-pyrrol-1-yl)-*1H*-pyrazol-1-yl)-*N,N*-dimethylethanamine: (contaminated with ca. 10% of regiomer): ^1^H NMR (CDCl_3_) δ: 7.57 (1H, s), 7.07-6.91 (4H, m), 6.84 (2H, d, J= 2.0 Hz), 6.25 (2H, d, J= 2.0 Hz), 4.21 (2H, t, J= 6.6 Hz), 2.83 (2H, d, J = 6.6 Hz), 2.32 (6H, s). ^13^C NMR (CDCl_3_) δ: 161.6 (d, ^1^J_CF_ =239 Hz), 145.4, 129.4, 129.1, 127.3, 120.7, 115.6, 113.5, 109.51, 58.7, 50.7, 46.4. HRMS (ESI, MH^+^) *m/z* 299.1666 (calcd for C_17_H_20_FN_4_ 299.1667).

**Protein expression and purification**

The stabilized DNA-binding domain of the p53 mutant Y220C, T-p53-Y220C, was expressed and purified as described previously. For the expression of ^15^N-labelled protein for NMR experiments, M9 minimal medium with ^15^NH_4_Cl (1 g/L) as the sole nitrogen source was used.

**
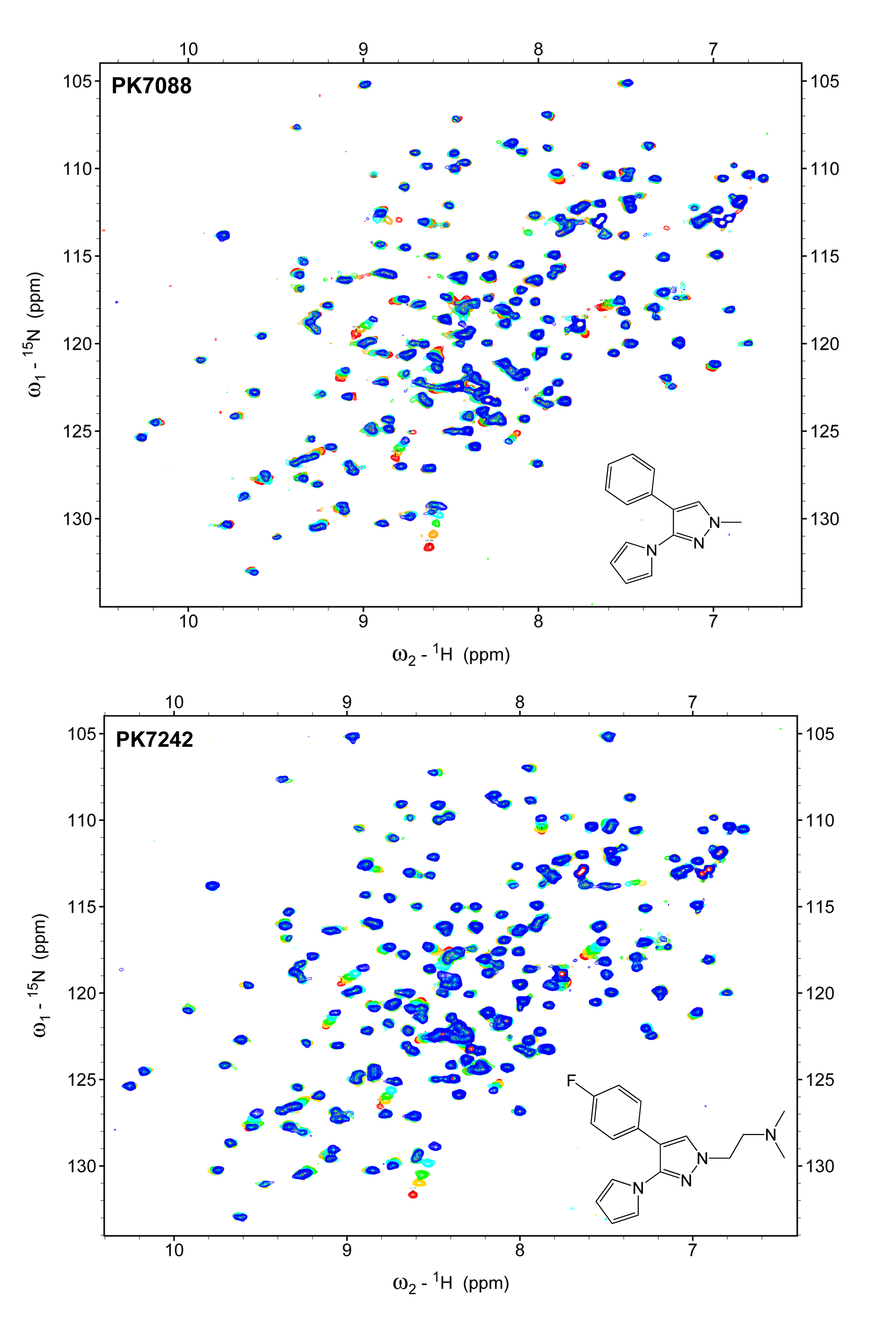
**

Figure S1. Overlay of ^1^H/^15^N-HSQC NMR spectra of p53-Y220C core domain (94-312) with varying concentrations of PK7088 (above) and PK7242 (below), indicating that both compounds have the same overall binding mode.

Figure S2. Relative fluorescence intensity after PK7088 treatment. The fluorescence intensity was calculated by ImageJ software.

Table S1. Kinase screen of PK7088 and its demethylated analogue PK7209.

| **Kinase** | **Activity in %**  **PK7088 @ 50 µM** | **Activity in %**  **PK7209 @ 50 µM** | **Study conducted by** |
| --- | --- | --- | --- |
|  |  |  |  |
| b-raf (h) | 110.2 | n.d. | CEREP S.A. |
|  |  |  |  |
| Abl(h) | 149 | 131 | Millipore |
| Abl(T315I)(h) | 128 | 106 | Millipore |
| Arg(h) | 109 | 98 | Millipore |
| Aurora-A(h) | 113 | 94 | Millipore |
| Bmx(h) | 99 | 95 | Millipore |
| BTK(h) | 85 | 83 | Millipore |
| CaMKIIβ(h) | 76 | 58 | Millipore |
| CaMKIV(h) | 79 | 76 | Millipore |
| CDK1/cyclinB(h) | 104 | 100 | Millipore |
| CDK2/cyclinA(h) | 97 | 100 | Millipore |
| CDK2/cyclinE(h) | 110 | 87 | Millipore |
| CDK3/cyclinE(h) | 86 | 87 | Millipore |
| CDK5/p35(h) | 74 | 78 | Millipore |
| CDK6/cyclinD3(h) | 89 | 89 | Millipore |
| CDK7/cyclinH/MAT1(h) | 89 | 79 | Millipore |
| CHK1(h) | 106 | 117 | Millipore |
| CHK2(h) | 113 | 92 | Millipore |
| CK1δ(h) | 90 | 60 | Millipore |
| CK1(y) | 64 | 53 | Millipore |
| CK2(h) | 111 | 86 | Millipore |
| CSK(h) | 103 | 107 | Millipore |
| c-RAF(h) | 89 | 83 | Millipore |
| cSRC(h) | 101 | 108 | Millipore |
| EGFR(h) | 123 | 137 | Millipore |
| EphB2(h) | 90 | 126 | Millipore |
| EphB4(h) | 101 | 117 | Millipore |
| FGFR3(h) | 107 | 106 | Millipore |
| Flt3(h) | 97 | 64 | Millipore |
| Fms(h) | 92 | 60 | Millipore |
| Fyn(h) | 84 | 83 | Millipore |
| GSK3β(h) | 101 | 71 | Millipore |
| IGF-1R(h) | 82 | 86 | Millipore |
| MAPK1(h) | 104 | 92 | Millipore |
| MAPK2(h) | 100 | 93 | Millipore |
| MEK1(h) | 103 | 95 | Millipore |
| Met(h) | 104 | 102 | Millipore |
| MST2(h) | 89 | 105 | Millipore |
| NEK2(h) | 106 | 106 | Millipore |
| p70S6K(h) | 98 | 69 | Millipore |
| PAK2(h) | 116 | 111 | Millipore |
| PAR-1Bα(h) | 100 | 87 | Millipore |
| PDGFRα(h) | 98 | 100 | Millipore |
| PDGFRβ(h) | 107 | 119 | Millipore |
| PDK1(h) | 96 | 95 | Millipore |
| PKA(h) | 112 | 111 | Millipore |
| PKBα(h) | 114 | 110 | Millipore |
| PKBβ(h) | 102 | 137 | Millipore |
| PKBγ(h) | 105 | 98 | Millipore |
| PKCα(h) | 92 | 90 | Millipore |
| PKCβII(h) | 82 | 81 | Millipore |
| PKCγ(h) | 97 | 81 | Millipore |
| PKCδ(h) | 103 | 89 | Millipore |
| PKCε(h) | 103 | 105 | Millipore |
| PKCη(h) | 108 | 101 | Millipore |
| PKCι(h) | 95 | 90 | Millipore |
| PKCμ(h) | 97 | 70 | Millipore |
| PKCθ(h) | 101 | 90 | Millipore |
| PKCζ(h) | 104 | 101 | Millipore |
| PKD2(h) | 105 | 89 | Millipore |
| PRAK(h) | 77 | 55 | Millipore |
| PRK2(h) | 98 | 84 | Millipore |
| Ros(h) | 99 | 106 | Millipore |
| Rsk1(h) | 65 | 55 | Millipore |
| Rsk1(r) | 100 | 58 | Millipore |
| Rsk2(h) | 91 | 49 | Millipore |
| Rsk3(h) | 94 | 59 | Millipore |
| SGK(h) | 136 | 120 | Millipore |
| Syk(h) | 108 | 123 | Millipore |
| Tie2 (h) | 96 | 86 | Millipore |
| Yes(h) | 106 | 114 | Millipore |

Table S2. X-ray data collection and refinement statistics

| *A. Data Collection* |  |
| --- | --- |
| Space Group | *P*2_1_2_1_2_1_ |
| Cell, a, b, c (Å) | 65.14, 71.02, 105.32 |
| Molecules/AU | 2 |
| Resolution (Å)^a^ | 29.6 - 1.35 (1.42 – 1.35) |
| Unique reflections | 107,334 |
| Completeness (%)^a^ | 99.7 (99.0) |
| Multiplicity^a^ | 5.4 (5.3) |
| R_merge_ (%)^a, b^ | 4.6 (54.5) |
| <I/σ_I_>^a^ | 17.6 (3.5) |
| Wilson B value (Å^2^) | 14.5 |
| *B. Refinement* |  |
| No. of non-hydrogen protein atoms^c^ | 3,206 |
| No. of non-hydrogen ligand atoms | 44 |
| No. of water molecules | 512 |
| No. of zinc atoms | 2 |
| R_cryst_, (%)^d^ | 15.5 |
| R_free_, (%)^d^ | 17.5 |
| R.m.s.d. bonds (Å) | 0.006 |
| R.m.s.d. angles (°) | 1.1 |
| Mean B value (Å^2^) | 19.5 |
| PDB entry | 3ZME |

^a^Values in parentheses are for the highest-resolution shell.

^b^R_merge_ = ∑(I_h,i_ - <I_h_>)/∑I_h,i_

^c^Number includes alternative conformations.

^d^R_cryst_ and R_free_ = ∑||F_obs_|- |F_calc_||/∑|F_obs_| where R_free_ was calculated over 5 % of the amplitudes chosen at random and not used in the refinement.
